# The Use of Nurse Checklists in a Bedside Computer-Based Information System to Focus on Avoiding Secondary Insults in Neurointensive Care

**DOI:** 10.5402/2012/903954

**Published:** 2012-07-15

**Authors:** Lena Nyholm, Anders Lewén, Camilla Fröjd, Tim Howells, Pelle Nilsson, Per Enblad

**Affiliations:** ^1^Department of Neuroscience/Neurosurgery, Uppsala University, 751 85 Uppsala, Sweden; ^2^Department of Surgical Sciences, Uppsala University, 751 85 Uppsala, Sweden

## Abstract

The feasibility and accuracy of using checklists after every working shift in a bedside computer-based information system for documentation of secondary insults in the neurointensive care unit were evaluated. The ultimate goal was to get maximal attention to avoid secondary insults. Feasibility was investigated by assessing if the checklists were filled in as prescribed. Accuracy was evaluated by comparing the checklists with recorded minute-by-minute monitoring data for intracranial pressure-ICP, cerebral perfusion pressure CPP, systolic blood pressure SBP, and temperature. The total number of checklist assessments was 2,184. In 85% of the shifts, the checklists were filled in. There was significantly longer duration of monitoring time at insult level when *Yes* was filled in regarding ICP (mean 134 versus 30 min), CPP (mean 125 versus 26 min) and temperature (mean 315 versus 120 min). When a secondary insult was defined as >5% of monitoring time spent at insult level, the sensitivity/specificity for the checklist assessments was 31%/100% for ICP, 38%/99% for CPP, and 66%/88% for temperature. Checklists were feasible and appeared relatively accurate. Checklists may elevate the alertness for avoiding secondary insults and help in the evaluation of the patients. This concept may be the next step towards tomorrow critical care.

## 1. Introduction

The importance of avoiding secondary brain insults, for example, high intracranial pressure (ICP), low cerebral perfusion pressure (CPP), and high temperature, after traumatic brain injury (TBI) was recognised in the 1970s [1]. This concept proved to be even more important for further improvements in outcome following the failure of the clinical trials with neuroprotective drugs [2]. To this end a secondary insult prevention program was introduced in the neurointensive care (NIC) unit at the department of neurosurgery in Uppsala in the 1990s [3]. Implementation of the secondary insult prevention program leads to a substantial improvement in outcome [3]. One cornerstone in the secondary insult program was the creation of a standardized management protocol system based on good laboratory practice (GLP) principles [4] that was developed and maintained by the doctors and nursing staff in a collaborative effort. Another cornerstone in the secondary insult program was the introduction of a new routine where the occurrence of secondary insults should be recorded in checklists by the nurses after every work shift. The assessments in the checklists should be reported to the next shift and at the clinical rounds. This strategy aimed to make all staff members maximally aware of their main task being to avoid secondary insult and to make it easy both for the doctors and the nurses to catch what the problems are for a certain patient. A bedside computer-based information system (QS patient data monitoring system, Version 6.8, General Electrics, Freiburg, Germany) was used by the nurses for the checklist recording. The assessment of the presence of secondary insults was based on criteria defined in the standardized management protocol system [3].

Our belief was that the use of checklists could be a valuable tool in improving the critical care by increasing the focus on the importance of avoiding secondary insults. Compared to the reports on the effects of checklists in aviation safety [5], very little have been written about effects within intensive care. The aims of this study were to evaluate the feasibility and accuracy of using nurse checklists integrated in a bedside computer-based information system for documentation of secondary insults with the ultimate goal to get maximal attention to avoid secondary insults in the neurointensive care (NIC) unit.

## 2. Material and Methods

### 2.1. Patient Characteristics on Admission

All consecutive patients older than 18 years with head injuries who had been monitored with ICP, CPP, and systolic blood pressure (SBP) for at least 7 days from 1 January 2008 to 31 October 2008 at the NIC Unit in Uppsala were identified and included in this study. A total of 91 patients were admitted with a head injury during this period. Twenty-six patients fulfilled the inclusion criteria. Thus, the study contained 26 patients, 21 men, and 5 women aged between 18 and 72 yrs (mean, 39 yrs). On admission to the NIC Unit, the patients were classified as Glasgow Coma Scale motor response (GCSM) 6–2 (mean 4.7). Demographic data were extracted from patient records.

### 2.2. Treatment of Traumatic Brain Injury at the NIC Unit

The patients were treated according to a standardized escalated management protocol [3, 6]. The goals were to keep ICP < 20 mm Hg and CPP around 60 mm Hg and to avoid all kinds of secondary insults. All patients who were not responding to commands (Glasgow Coma Scale Motor response GCSM ≤ 5) were intubated and were artificially ventilated. Moderate hyperventilation with a pCO_2_ 4.0–4.5 kPa was initially applied, but gradually adjusted towards normoventilation under surveillance of ICP. The artificially ventilated patients received propofol and morphine chloride. The reaction level was checked regularly. ICP monitoring was considered to be indicated in all patients not responding to commands (GCSM ≤ 5). A ventricular drainage system was used if possible (Smiths medical, Grasbrunn, Germany), but in cases with a compressed ventricular system a parenchymal probe was used instead (Codman ICP express, Johnson & Johnson, Raynham, USA). The patients' heads were slightly elevated to facilitate venous outflow. Significant mass lesions were evacuated. If ICP remained elevated despite this basal treatment, cerebrospinal fluid drainage, Pentothal coma treatment, and external decompressive craniectomy were used in an escalated order.

### 2.3. Standardized Management Protocol System—Treatment Goals

The standardized management protocol system developed at the NIC Unit in Uppsala is based on the GLP principles and contains written instructions that describe all kinds of routines, that is, standard operating procedures (SOP) [4]. The main objective is to make all staff members maximally aware that their main task is to avoid secondary insult. Therefore, treatment goals have been defined (Table 1).

### 2.4. Secondary Insult Checklist

**Figure 1:**
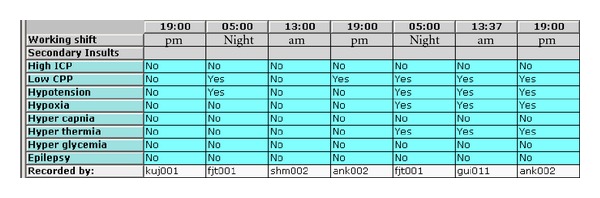
The checklist recording of secondary insults in a bedside computer-based information system. This figure shows, for example, that low CPP was a significant problem during five shifts.

Nurses at the NIC Unit in Uppsala work in 3 shifts, 07:00–14:00, 14:00–21:00, and 21:00–07:00. After every work shift, the nurses should record if there had been any secondary insults or not during their shift by ticking a box for *Yes* or *No* for each of 8 insult categories in a checklist in the bedside computer-based information system (Figure 1). According to the standardized procedure, presence of secondary insult should be recorded if all regular treatment procedures outlined in the standardized management system have been performed and the patient still has not reached the treatment goals (Table 1). It could not be defined exactly in the standardized instruction when insults should be assessed to have occurred since the patterns may look very different, for example, high values during a very short continuous period of time, values close to goal during a long continuous period of time, or scattered values at insult level. Instead, the overall impression of whether the patient reached the treatment goals or not according to the nurse's clinical experience was applied. This approach was found feasible since the main purpose was to increase the awareness for secondary insults. The assessments in the checklist should be viewed upon as a summary review of the occurrence of secondary insults for the ongoing nurse to highlight the problems during the shift before.

### 2.5. Monitoring Data and Quantification of Secondary Insults

The Odin monitoring system developed by Tim Howells and colleagues was used for collection and retrospective analysis of minute-by-minute monitoring data [7]. In this study, data from ICP, CPP, systolic blood pressure (SBP), and temperature from the first 7 days of monitoring were extracted. The quality of the monitoring data was screened and clear artefacts removed using the Odin software. The monitoring time left after artefact removal and exclusion of gaps in monitoring data associated with, for example, radiology examinations or surgical procedures was defined as good monitoring time (GMT). The amount of secondary insults was calculated as the proportion of GMT spent above/below defined insult levels for ICP, CPP and temperature. When good monitoring time (GMT) was calculated for the 26 patients, 5 had to be excluded due to technical problems analysing the monitoring files of those patients.

### 2.6. Investigation of the Feasibility and Accuracy of Using Secondary Insult Checklists

The *feasibility* of using checklists was evaluated by counting to which extent the checklists were filled in as prescribed by the standardized management guide line protocol (Table 2).

The *accuracy* of using checklists was evaluated in four different ways by comparing the checklist assessments with the actual occurrence of secondary insults according to the collected minute-by-minute monitoring data (Table 2). (1) The proportions of *Yes* and *No* in assessed work shifts with no collected minute-by-minute values out of the treatment goal were calculated; (2) the duration in minutes spent at secondary insult level was compared between *Yes* and *No* assessments in assessed work shifts with any value out of the treatment goal; (3) the numbers of *Yes and No *were analysed in relation to the proportions of GMT spent above/below the defined insult level for all work shifts; (4) the sensitivity and specificity for the checklist assessments were calculated. A secondary insult was defined to have occurred if >5% of GMT had been spent at insult level according to the collected minute-by-minute monitoring data.

The reason why the comparison between the checklist assessments and the monitoring data was done in four different ways was because no golden standard exists how to summarize monitoring data and the occurrence of secondary insults although the proportion of GMT spent at insult level is widely used [8]. The study outline and selections made are presented in Table 2.

### 2.7. Statistical Methods

*t* tests were performed to detect differences between checklist assessment (*Yes/No*) of secondary insults and actual occurrence of secondary insults according to the min-by-min monitoring data. Statistical significance was set to *P* < 0.05. Furthermore, the sensitivity and specificity was calculated for the checklists. In the calculations of sensitivity and specificity, a secondary insult was considered to have occurred if >5% of the GMT did not reach the treatment goals.

## 3. Results

### 3.1. Nurses' Documentation of Secondary Insults

The study contained 546 work shifts where assessments regarding secondary insults (ICP, CPP, SBP, and temperature) should have been conducted by nurses. The total number of assessments was 2184. The nurses documented their assessments in 84-85% of their shifts: ICP 84%, CPP 84%, SBP 84%, and temperature 85%. High temperature was documented in 28% (155/546) of the shifts, high ICP in 13% (70/546), low CPP in 8% (41/546), and low SBP in 2% (13/546) of the shifts.

### 3.2. Correspondence between the Nurses' Documentation of Secondary Insults and Monitored Values

Analysis of shifts with no monitored values out of the treatment goals showed that 776 of 803 assessments (97%) were correctly documented as *No* secondary insult, and 27 (3%) were incorrectly documented as *Yes* for secondary insult. Twenty-four of the wrong registrations concerned temperature.

Analysis of shifts with any monitored values out of the treatment goals showed statistically significantly longer durations of minutes above/below threshold for ICP, CPP, and temperature when *Yes* was documented for the occurrence of secondary insults (Table 3). Concerning SBP, there were no significant differences for duration below threshold between *Yes* and *No* assessments (Table 3).

**Figure 2 fig2:**
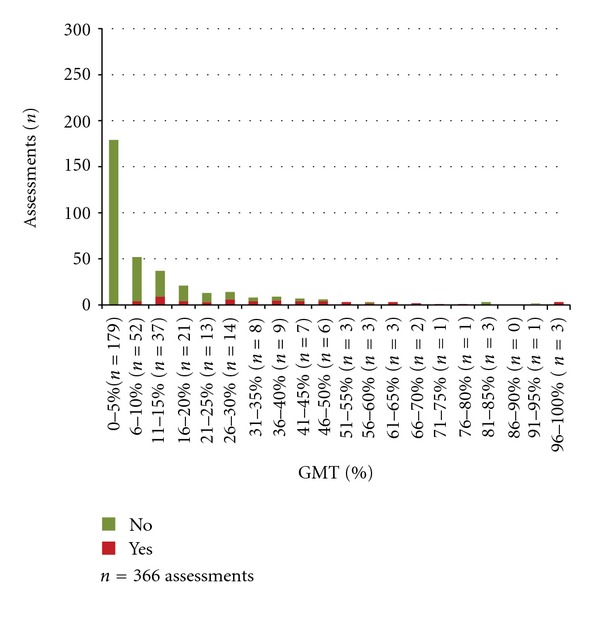
Percent of GMT with ICP > 20 mm Hg and the nurses' assessments of secondary insults.

**Figure 3:**
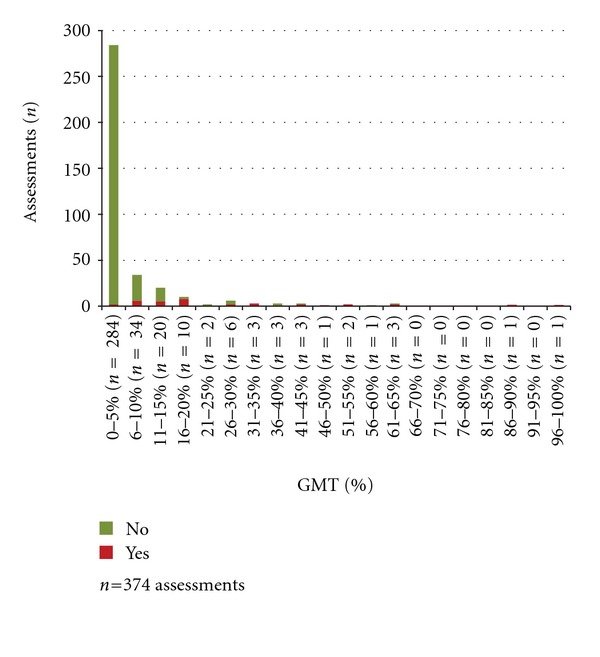
Percent of GMT with CPP < 60 mm Hg and the nurses' assessments of secondary insults.

**Figure 4 fig4:**
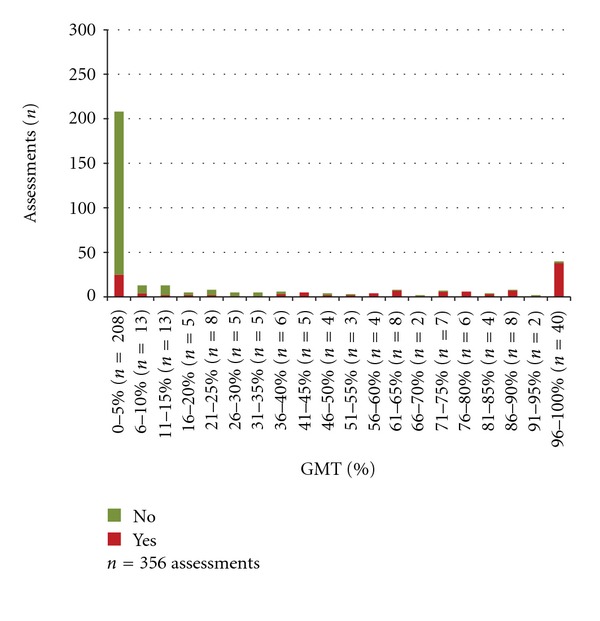
Percent of GMT with temperature > 38°C and the nurses' assessments of secondary insults.

The results of the nurses' checklist assessments in relation to the proportion (%) of GMT spent above/below insult levels for ICP, CPP, and temperature are presented in Figures 2, 3, and 4. The nurses' assessments in relation to if >5% of GMT was spent at insult level are presented in Table 4. When a secondary insult was defined to have occurred if >5% of GMT was spent at insult level, the sensitivity was calculated to 31% for ICP, 38% for CPP, and 66% for temperature (Table 4). The specificity was 100% for ICP, 99% for CPP, and 88% for temperature (Table 4).

## 4. Discussion

The NIC nurse was identified as the key person responsible for reducing the occurrence of secondary insults [9, 10]. The nurse checklists for the occurrence of secondary insults were introduced as part of a secondary insult program initiated at our NIC Unit with the hope that maximal attention should be paid on avoiding secondary insults and that the checklists should facilitate quick evaluation of the patients [3]. Earlier evaluation of the secondary insult programme which also included establishment of a standardized management protocol system showed substantially improved results [3]. It is difficult to evaluate objectively to which extent the improvement could be ascribed to the use of the checklists. The subjective impression was clearly that the checklists had a positive influence on the management of the patients and facilitated the evaluation of the patients. This study is an attempt to evaluate the feasibility and accuracy of using secondary insult nurse checklists in NIC unit in a bedside computer-based information system.

### 4.1. Feasibility of Computer-Based Checklist Nurse Recording of Secondary Insults

The working conditions in critical care are usually intensive and unpredictable due to the severe conditions of the patients and the advanced management required with short notice of time. The main focus is on life-saving procedures, and, even if important, documentation is a secondary task. The principles for routine documentation need to be straight and simple to be useful. The objective of documenting certain information must also be obvious to provide motivation. The introduction of nurse checklist recording of the occurrence of secondary insults after every shift inevitably increases the workload. The finding that the nurses conducted their documentation in as much as 85% of the occasions during NIC conditions indicates that computer-based checklist nurse recording of secondary insults was feasible in neurointensive care and that the purpose was clear, that is, to devote maximal attention to reducing secondary insults and reduce secondary brain injury. The usefulness and feasibility of computer-based checklist nurse recording of secondary insults may also be reflected in the validity of registration. Therefore, it is also important to compare the checklist registrations with the actual occurring insults according to the collected minute-by-minute monitoring data.

### 4.2. The Accuracy of Checklist Nurse Recording of Secondary Insults

ICP, CPP, and SBP were monitored ≥90% of the time in 87% of the work shifts assessed, and temperature was monitored ≥90% of the time in 60% of the work shifts which provides a substantial amount of actual data to compare the checklists with (data not presented). The correspondence between the checklist registrations of secondary insults and the actual monitoring values collected was evaluated in different ways. When the shifts without monitoring values out of the treatment goals were analysed, only 27 of 776 *Yes* boxes were ticked (occurrence of secondary insult) and 24 of these positive assessments concerned temperature. The positive temperature assessments are probably explained by the fact that temperature is sometimes measured intermittently in the axilla and that those data are not stored. However, overall, this comparison indicates high accuracy of the checklist assessments.

When the checklist assessment and the mean duration of minutes at insult level were compared, the mean duration of values out of the goal was short for ICP, CPP, and SBP while for temperature the mean duration was clearly longer. The mean duration of insults showed a statistically significant difference between *Yes* and *No* for ICP, CPP, and temperature (Table 2). No significant difference was found for SBP but the proportion of *Yes* was small, and the duration of values out of the goal was very short (Table 2). It is also apparent in this analysis that the checklist assessments were relatively accurate. It should also be emphasised that it was not possible to define exactly in the standardized instruction when insults should be assessed to have occurred during a shift since the patterns may look very different, for example, high values during a very short continuous period of time, values close to goal during a long continuous period of time, or scattered values at insult level. Instead, the overall impression of whether the patient reached the treatment goals or not according to the nurse's clinical experience that was applied.

When the burden of insults is quantified, the proportion of GMT spent at insult level is frequently used as a summary measure [8]. The advantage of this measure is that it is influenced both by the duration and the degree of the insult although it does not show whether there was a long period with values, close to the goal, a short period with very high/low values or scattered insult values. Comparing the checklist assessments and the proportion of GMT at insult level shows a clear pattern where the proportion of *Yes* ticks is increased by increasing percent of GMT spent at insult level (Figures 2–4).

For the specificity and sensitivity calculations, 5% of GMT spent at insult level was used as a cutoff to decide if a secondary insult had taken place. The result shows that there is a good specificity and a poor sensitivity for the checklist assessment of secondary insults, that is, high probability of *No* if secondary insults had not occurred and low probability of *Yes* if secondary insults occurred. In other words, by using the assessment, it is easier to correctly identify a true absence of secondary insults than to correctly identify a true presence of secondary insults. A cutoff of 5% is low and taking into consideration that many insult values may also be close to the insult threshold and scattered over time, and this result indicates that the assessment is clinically relevant.

## 5. Conclusion

The present study showed that checklists integrated in a bedside computer-based information system were feasible to use under NIC conditions. The checklist assessment of secondary insults appeared to be relatively accurate and clinically relevant. This study may serve as an example of how computer-based checklists can be used in combination with a standardized management protocol system to improve the management of critically ill patients in intensive care units. We believe that the introduction of standardized checklists elevates the alertness for avoiding secondary insults and helps in the evaluation of the patients. This concept may be the next step towards tomorrow's critical care.

## Figures and Tables

**Table 1:** Treatment goals according to the standardized management protocol system.

Treatment goals
ICP <20 mm Hg
CPP >60 mm Hg
SBP >100 mm Hg
pO_2_ >12 kPa
pCO_2_ 4.0–4.5 kPa
Temperature <38^°^C
Blood glucose 5–10 mmol/L

ICP: intracranial pressure.

CPP: cerebral perfusion pressure.

SBP: systolic blood pressure.

**Table 2:** Study design outline.

Evaluation of secondary insult checklists	Measures	Study material/selections	Table or figure
Feasibility	The extent of filled in checklists	All shifts in 26 patients. 2184 assessments (ICP 546, CPP 546, SBP 546, and temperature 546).	

Accuracy	(1) The proportions of *Yes* and *No* assessments in shifts with no collected minute-by-minute values out of the treatment goal	Assessed work shifts with complete monitoring data and no collected minute-by-minute values out of the treatment goal in 26 patients. 803 assessments (ICP 58, CPP 179, SBT 320, and temperature 246)	
	(2) The duration in minutes spent at secondary insult level compared to how the assessment was made (*Yes* or *No*) in shifts with any value out of the treatment goal	Assessed work shifts with complete monitoring data and any value out of the treatment goal in 26 patients. 929 assessments (ICP 381, CPP 260, SBP 129, and temperature 159)	Table 3
	(3) The numbers of *Yes* and *No* assessments in relation to the proportions of GMT spent above/below the defined insult level.	Assessed work shifts with complete monitoring data in 21 patients^∗^. 1096 assessments (ICP 366, CPP 374, and temperature 356)	Figures 2–4
	(4) The sensitivity and specificity for the checklist assessments. A secondary insult was defined to have occurred if >5% of GMT had been spent at insult level according to the collected minute-by-minute monitoring data.	Assessed work shifts with complete monitoring data in 21 patients^∗^. 1096 assessments (ICP 366, CPP 374, temperature 356)	Table 4

^∗^5 patients had to be excluded due to technical problems analysing the monitoring files.

**Table 3:** Duration in minutes spent at secondary insult level in relation to *Yes* or *No* assessment for assessed work shifts with complete monitoring data and any value out of the treatment goal.

Variable	Checklist assessment *Yes* (*n*)	Mean duration (min/SD)	Checklist assessment *No* (*n*)	Mean duration (min/SD)	*P* value
ICP >20 mm Hg	64	134/111	317	30/47	<0.001
CPP <60 mm Hg	37	125/110	223	26/44	<0.001
SBT <100 mm Hg	10	19/26	119	9/25	0.6
Temperature >38°C	104	315/166	55	120/111	<0.001

ICP: intracranial pressure.

CPP: cerebral perfusion pressure.

SBP: systolic blood pressure.

**Table 4:** Sensitivity and specificity for the checklist assessments.

Checklist assessment	ICP		CPP		Temperature	
	Yes	No	Yes	No	Yes	No
>5% of GMT	58^1^	129^4^	34^1^	56^4^	97^1^	51^4^
	(16%)	(35%)	(9%)	(15%)	(27%)	(14%)

<5% of GMT	0^3^	179^2^	2^3^	282^2^	25^3^	183^2^
		(49%)	(1%)	(75%)	(7%)	(52%)

Sensitivity	31%		38%		66%	
>5% of GMT	(58^1^/187^1+4^ = 0.31)		(34^1^/90^1+4^ = 0.38)		(97/148^1+4^ = 0.66)	

Specificity	100%		99%		88%	
<5% of GMT	(179^2^/179^2+3^ = 1.0)		(282^2^/284^2+3^ = 0.99)		(183^2^/208^2+3^ = 0.88)	

A secondary insult was defined to have occurred if >5% of GMT had been spent at insult level according to the collected minute-by-minute monitoring data.

(1) Number of true positive checklist assessments.

(2) Number of true negative checklist assessment.

(3) Number of false positive checklist assessments.

(4) Number of false negative checklist assessments.

ICP: intracranial pressure.

CPP: cerebral perfusion pressure.
